# Nitrogen Cycling Microbial Diversity and Operational Taxonomic Unit Clustering: When to Prioritize Accuracy Over Speed

**DOI:** 10.3389/fmicb.2022.730340

**Published:** 2022-05-26

**Authors:** Sada Egenriether, Robert Sanford, Wendy H. Yang, Angela D. Kent

**Affiliations:** ^1^Program in Ecology, Evolution and Conservation Biology, University of Illinois at Urbana-Champaign, Urbana, IL, United States; ^2^Department of Geology, University of Illinois at Urbana-Champaign, Urbana, IL, United States; ^3^Department of Plant Biology, University of Illinois at Urbana-Champaign, Urbana, IL, United States; ^4^Department of Natural Resources and Environmental Sciences, University of Illinois at Urbana-Champaign, Urbana, IL, United States

**Keywords:** bioinformatics, mother, nitrogen cycling, microbiome, nitrogen fixation, dissimilatory nitrate reduction to ammonium, OTU clustering, microbial ecology

## Abstract

**Background:**

Assessments of the soil microbiome provide valuable insight to ecosystem function due to the integral role microorganisms play in biogeochemical cycling of carbon and nutrients. For example, treatment effects on nitrogen cycling functional groups are often presented alongside one another to demonstrate how agricultural management practices affect various nitrogen cycling processes. However, the functional groups commonly evaluated in nitrogen cycling microbiome studies range from phylogenetically narrow (e.g., N-fixation, nitrification) to broad [e.g., denitrification, dissimilatory nitrate reduction to ammonium (DNRA)]. The bioinformatics methods used in such studies were developed for 16S rRNA gene sequence data, and how these tools perform across functional genes of different phylogenetic diversity has not been established. For example, an OTU clustering method that can accurately characterize sequences harboring comparatively little diversity may not accurately resolve the diversity within a gene comprised of a large number of clades. This study uses two nitrogen cycling genes, *nifH*, a gene which segregates into only three distinct clades, and *nrfA*, a gene which is comprised of at least eighteen clades, to investigate differences which may arise when using heuristic OTU clustering (abundance-based greedy clustering, AGC) vs. true hierarchical OTU clustering (Matthews Correlation Coefficient optimizing algorithm, Opti-MCC). Detection of treatment differences for each gene were evaluated to demonstrate how conclusions drawn from a given dataset may differ depending on clustering method used.

**Results:**

The heuristic and hierarchical methods performed comparably for the more conserved gene, *nifH*. The hierarchical method outperformed the heuristic method for the more diverse gene, *nrfA*; this included both the ability to detect treatment differences using PERMANOVA, as well as higher resolution in taxonomic classification. The difference in performance between the two methods may be traced to the AGC method’s preferential assignment of sequences to the most abundant OTUs: when analysis was limited to only the largest 100 OTUs, results from the AGC-assembled OTU table more closely resembled those of the Opti-MCC OTU table. Additionally, both AGC and Opti-MCC OTU tables detected comparable treatment differences using the rank-based ANOSIM test. This demonstrates that treatment differences were preserved using both clustering methods but were structured differently within the OTU tables produced using each method.

**Conclusion:**

For questions which can be answered using tests agnostic to clustering method (e.g., ANOSIM), or for genes of relatively low phylogenetic diversity (e.g., *nifH*), most upstream processing methods should lead to similar conclusions from downstream analyses. For studies involving more diverse genes, however, care should be exercised to choose methods that ensure accurate clustering for all genes. This will mitigate the risk of introducing Type II errors by allowing for detection of comparable treatment differences for all genes assessed, rather than disproportionately detecting treatment differences in only low-diversity genes.

## Introduction

Microbial community structure is important to characterize because it can influence many ecosystem processes (Graham et al., 2016). For assessments of overall microbial community composition, the 16S rRNA gene is typically used because it is highly conserved across prokaryotes and generally not subject to horizontal gene transfer. Given the ubiquity of 16S rRNA assessments within microbiome research, the bioinformatics pipelines used to process raw amplicon sequence data were developed with a focus on 16S rRNA gene sequence data specifically. Over the last decade, the approaches used to preprocess sequences, cluster unique sequences into OTUs, and assign taxonomic classification have been continually expanding and improving. However, side-by-side comparisons of 16S rRNA datasets resulting from pipelines which differ in only one or two key steps have demonstrated that upstream processing decisions (e.g., clustering of sequences) can influence conclusions about differential abundance, composition, taxonomic identity, and richness and diversity measures (Chen et al., 2013; Nguyen et al., 2016; López-García et al., 2018).

As a complement to overall community composition, studies focusing on specific microbially mediated ecological processes often characterize functional groups relevant to the process being investigated. These functional groups fall along a diversity spectrum ranging from processes which are performed by a comparatively narrow selection of taxa, or phylogenetically “narrow” processes, to those which can be performed by a large variety of taxa, or “broad” processes (Schimel and Schaeffer, 2012). The same bioinformatics tools developed for 16S rRNA gene sequences are used to process diagnostic gene sequences for these types of functional groups as well. Though efforts have been made to identify discrepancies among results generated from different processing methods using 16S rRNA gene sequence datasets, the impact of processing choices on downstream community analyses for functional genes of varying diversity has not yet been explored.

For both 16S rRNA and functional genes, amplicon sequence data are often generated *via* Illumina sequencing in the form of millions of paired-end reads. Generally, primers for amplicon sequencing are designed to generate forward and reverse reads which overlap and can be assembled into continuous sequences, or contigs. The first step in processing raw sequence data for downstream OTU clustering is therefore merging the two reads and filtering the resulting contigs for quality control. Modern sequencing instruments return a quality score for each base in each sequence, and this quality score, together with agreement between bases in the overlapping portion of paired reads, is used to filter out low-quality contigs. These steps can be achieved using many common bioinformatics software packages, including Usearch (Edgar, 2013), FLASH (Magoè and Salzberg, 2011), Mothur (Schloss et al., 2009), or QIIME (Caporaso et al., 2010). The implementation of these steps is similar among all packages and allows the user to provide arguments to customize quality cutoffs as desired. The end result of this stage of preprocessing is a list of all unique sequences which passed quality filtering.

After quality screening, unique sequences are assigned to an operational taxonomic unit (OTU), which is most often achieved by clustering sequences according to some similarity percentage. The purpose of clustering is twofold: clustering sequences together by similarity helps to eliminate erroneous sequences formed during the PCR preamplification step carried out prior to sequencing, as each of these erroneous sequences should deviate from one another by only a few bases, thus reducing diversity to true biological diversity (Hugerth and Andersson, 2017). In addition, collapsing the full breadth of sequence diversity into groups within some percentage similarity of one another reduces the total number of “variables” in downstream analyses, making them more computationally tractable.

The similarity threshold typically chosen is 97% (Gevers et al., 2005), as similarity levels lower than this in the 16S rRNA gene region are considered unlikely to be derived from the same species and unlikely to achieve 70% DNA-DNA hybridization at the genome level, a previously common metric for determining bacterial species assignment (Gevers et al., 2005). This, however, can only evaluate the gene region considered, and does not necessarily reflect 97% similarity across the full length of the gene. This is also an arbitrary cutoff, as individual taxa may possess 16S rRNA genes that are more than 97% similar but still represent ecologically distinct clades based on the remainder of their genome content (Fox et al., 1992; Gevers et al., 2005). As sequencing throughput and quality has increased in recent years, a 99% similarity cutoff for inclusion in an OTU has become increasingly common (Hugerth and Andersson, 2017). Acceptable percent similarity cutoffs for OTUs generated from functional gene sequences have not yet been established, and 97% is still typically used, regardless of the diversity within the gene’s phylogeny.

Clustering approaches can be reference-based or *de novo*. The former uses a reference taxonomic database to classify sequences into taxonomic bins based on known taxonomy, while the latter allows the data to “speak for themselves” by assigning sequences to clusters based on similarity alone (Schloss and Westcott, 2011). Reference-based clustering can be either closed reference, wherein sequences are mapped to their best possible match within a database, and those that do not match sufficiently are discarded, or open reference, where those sequences which do not match to the reference are then clustered using the *de novo* approach. In *de novo* clustering, approaches may be hierarchical (based on single, average, or complete linkage) (Schloss and Handelsman, 2005) or heuristic in strategy. Single-linkage hierarchical approaches place a sequence into a cluster if it has a similarity above some threshold to at least one other sequence in the cluster, while complete linkage conversely requires a sequence to have a similarity above the threshold to *all* others in the cluster; average linkage requires that the average similarity between a sequence and all others be above the threshold (Hugerth and Andersson, 2017). Average-linkage *de novo* clustering has been demonstrated to produce higher quality OTUs based on the Matthew’s Correlation Coefficient (MCC), a metric for describing the ratio of False Positives (FP), False Negatives (FN), True Positives (TP), and True Negatives (TN) commonly used in machine learning control theory (Westcott and Schloss, 2015). However, because it is computationally expensive to run all-against-all comparisons on datasets containing millions of reads, heuristic approaches were developed. Among these are the UPARSE algorithm implemented *via* USEARCH (Edgar, 2010), which approximates average-linkage approaches by comparing a sequence to only one centroid sequence within each cluster. The choice between true hierarchical clustering and heuristic clustering therefore represents a tradeoff between computational speed and accuracy.

The distribution of distances between sequences in clusters will differ depending on the clustering approach used, even if using the same similarity cutoff (Hugerth and Andersson, 2017). Among commonly used modern tools, hierarchical clustering is available through the “Opti-MCC” method implemented in Mothur (Westcott and Schloss, 2017), which is now included as the default clustering method. Heuristic approaches are available through USEARCH (Edgar, 2013), QIIME (Caporaso et al., 2010), which runs Uclust in the background, and VSEARCH (Rognes et al., 2016), an open-source alternative to USEARCH. Among these heuristic options, abundance-based greedy clustering (AGC) is often the default implementation. The AGC clustering algorithm begins with the most abundant unique sequences in the dataset and begins building OTUs from these. This operates under the assumption that the most abundant sequences are more likely to be biologically “accurate,” and do not represent sequencing errors. That the AGC method is “greedy” means that as it works through the list of sequences, it places a sequence with the first match it finds that meets the percent similarity threshold—it does not continue looking to see if there is an even closer match, and once a decision is made, it cannot be changed afterward. The implication of this is that resulting clusters are less accurate, and thus often leads to fewer OTUs and more dissimilar sequences assigned to each OTU, despite taking far less time to compute. In addition, this approach may introduce spurious correlations between samples or treatments which are overrepresented in the most abundant sequences. Conversely, Opti-MCC, a hierarchical clustering method, uses an iterative approach which repeatedly reevaluates the clusters formed until the MCC (ratio of FP, FN, TP, TN) (Westcott and Schloss, 2015) is optimized. This method begins with each unique sequence as its own OTU, and checks whether combining each pair of OTUs will improve the MCC—if it does, they are combined. This progresses until no further combinations remain that will improve the MCC. Unsurprisingly, this iterative approach requires much more time to execute, but the results are optimized clusters which more accurately group sequences according to percent similarity. Therefore, the choice of clustering approach can affect downstream analyses and conclusions regarding microbial community diversity and structure due to its direct effect on assignment of sequences to OTUs.

To further complicate matters, many common multivariate analyses used in microbiome studies are sensitive to uneven count data (unequal number of sequence reads per sample), thus requiring normalization prior to analysis. Historically, this has been achieved through rarefaction, which involves randomly subsampling each sample’s reads down to an even depth. This, however, has been demonstrated to reduce statistical power, and may dramatically change the conclusions drawn from downstream multivariate analyses like PERMANOVA (McMurdie and Holmes, 2014). Comparing analytical results from repeated rarefying trials has been previously suggested (Navas-Molina et al., 2013), but this is time consuming and is not generally practiced. Therefore, most published results are obtained from analyses performed on a rarefied OTU table which was produced by randomly subsampling the original OTU table a single time. When the entire burden of proof for downstream analyses rests on this single subsample, consistency among random samples becomes critically important. Otherwise, we allow chance to determine whether or not our subsample contains enough statistical power to reject the null hypothesis. For differential abundance analyses focusing on individual OTUs, we are able to sidestep these pitfalls of rarefaction by using the negative binomial mixed model implementation in R package DESeq2 (Anders and Huber, 2010); however, this approach still depends on the accuracy of the upstream OTU clustering. Ultimately, regardless of the analysis methods used downstream, the accuracy of OTU assignment will play a role in interpreting DNA sequence data.

These major considerations and shortcomings related to OTU clustering for 16S rRNA gene sequences, the most commonly sequenced gene for which all of these methods were developed, do not even begin to address methodological considerations for the quagmire of diversity found within common functional genes of interest. For example, the suite of functional genes commonly evaluated in microbiome studies concerning nitrogen (N) cycling range from phylogenetically narrow (e.g., N-fixation, nitrification) to broad [e.g., denitrification, dissimilatory nitrate reduction to ammonium (DNRA)]. However, a method which can accurately characterize sequences harboring comparatively little diversity (which can be binned into fewer OTUs) may not accurately resolve the diversity within a gene comprised of a large number of clades. The diagnostic gene for N-fixation, *nifH*, segregates into only 3 distinct clades (Raymond et al., 2004), and thus an algorithm like AGC can be expected perform sufficiently because each of these clades are likely to be represented within the most abundant sequences. In contrast, the diagnostic gene for DNRA, *nrfA*, is comprised of 18 distinct clades (Welsh et al., 2014). In this case, an abundance-based method which preferentially assigns sequences to the largest clusters runs the risk of pulling sequences that might otherwise represent their own smaller clusters into the larger initial clusters. Because AGC is also “greedy,” this means that there is no reassessment afterward to reconcile this error. A true hierarchical clustering method, however, will be more likely to detect these smaller clusters of similarity and assign them to their own OTU as clustering proceeds. Since functional gene community analyses are typically presented together to illustrate treatment effects on specific functional groups, these differences in accuracy may introduce biases in the conclusions that may be drawn from the resulting OTU datasets.

In this study, we evaluated the performance of two clustering methods, one heuristic (AGC) and one hierarchical (Opti-MCC), on a functional gene comprised of few clades (*nifH*) and a gene comprised of many clades (*nrfA*). We predicted that both the AGC and Opti-MCC methods would perform similarly on *nifH* sequences, but that Opti-MCC would produce more statistical power than AGC when applied to *nrfA* sequences due to its greater ability to characterize the diversity within the gene. We processed the same two sets of raw Illumina sequences in Mothur, once using the Opti-MCC clustering algorithm at the standard 97% similarity cutoff, and twice using the AGC algorithm: Once at 97% similarity, and again at 98%, to evaluate if tightening the similarity threshold was able to better resolve greater diversity. Each resulting OTU table was then subjected to 10 repeated rarefaction trials, which generated 10 rarefied OTU tables from each raw OTU table. The ability to detect community differences using common multivariate methods was assessed for each rarefied dataset and results were aggregated for comparison between methods. Alpha diversity metrics were also assessed for each unrarefied and rarefied dataset. Finally, differential abundance analyses were conducted on each original unrarefied OTU table to evaluate performance independent of rarefaction, as well as performance in taxonomic classification. Based on these comparisons, we conclude with recommendations about appropriate use cases for each method, and when priority should be placed on clustering accuracy vs. computational speed.

## Materials and Methods

### Data Source

The DNA sequence data used in this study are part of a larger dataset used to compare soil microbial communities among agricultural management treatments. The dataset derives from DNA extracted from soils collected from several fields at the University of Illinois Crop Sciences Research and Education Center located in Urbana, Illinois (40°03′32.0″N 88°13′34.0″W), representing four agricultural treatment groups, with *n* = 8 for each group. This dataset is intended to simulate a “real world” application of processing and downstream analysis, and to highlight actual differences in conclusions and interpretation that might be impacted by upstream processing choices. While intentionally assembled mock communities enable performance evaluation against a known assemblage of taxa, methods which perform well on these simulated communities often do not perform well when faced with the phylogenetic diversity encountered in environmental samples (Westcott and Schloss, 2015). For this study, treatments are generically abbreviated as T1 through T4, where T1 and T2 = conventionally tilled corn-soy rotation (separate sites), T3 = no till corn-soy rotation, and T4 = perennial grasses (Miscanthus and switchgrass).

### DNA Extraction and Molecular Methods

Total genomic DNA was extracted from freeze-dried soil samples using the FastDNA SPIN Kit for Soil (MP Biomedicals, Solon, OH). Genomic DNA was further purified using a cetyl trimethyl ammonium bromide (CTAB) extraction to remove contaminating humic acids. DNA concentration was adjusted to a standard concentration of 10 ng/μL in each sample.

Illumina sequencing was used to target nitrogen cycling functional genes *nifH* and *nrfA* (Illumina, San Diego, CA). Additional primer details can be found in Supplementary Table 1. Sequencing amplicons were prepared by PCR using a Fluidigm Access Array IFC chip, which allowed simultaneous amplification of each target gene (Fluidigm, San Francisco, CA). Initial reactions were carried out according to a 2-step protocol using reagent concentrations according to Fluidigm parameters. The first PCR was performed in a 100-μL reaction volume using 1 ng DNA template. This PCR amplified the target DNA region using both the gene-specific primers with Fluidigm-specific amplification primer pads CS1 (5′-ACACTGACGACATGGTTCTACA-3′) and CS2 (5′-TACGGTAGCAGAGACTTGGTCT-3′), which produced amplicons including (1) CS1 Fluidigm primer pad, (2) 5′-forward PCR primer, (3) amplicon containing the region of interest, (4) 3′-reverse PCR primer, and (5) CS2 Fluidigm primer pad. A secondary 30-μL PCR used 1 μL of 1:100 diluted product from the first PCR as template, and added Illumina-specific sequencing linkers P5 (5′-AATGATACGGCGACCACCGAGATCT-3′) and P7 (5′-CAAGCAGAAGACGGCATACGAGAT-3′), along with a 10-bp sample-specific barcode sequence. The final construct consisted of (1) Illumina linker P5, (2) CS1, (3) 5′-primer, (4) amplicon containing the region of interest, (5) 3′-primer, (6) CS2, (7) sample-specific 10-bp barcode, and (8) the Illumina linker P7. Final amplicons were gel-purified, quantified (Qubit; Invitrogen, Carlsbad CA, United States), combined to the same concentration, and then sequenced from both directions on an Illumina HiSeq 2500 2 × 250 bp Rapid Run. Fluidigm amplification and Illumina sequencing was conducted at the Roy J. Carver Biotechnology Center (Urbana, IL, United States). Barcodes were used to assign each sequence to its original sample, and sequences were provided as demultiplexed.fastq sequences with adaptors, barcodes, and primer sequences removed.

### Experimental Design

**FIGURE 1:**
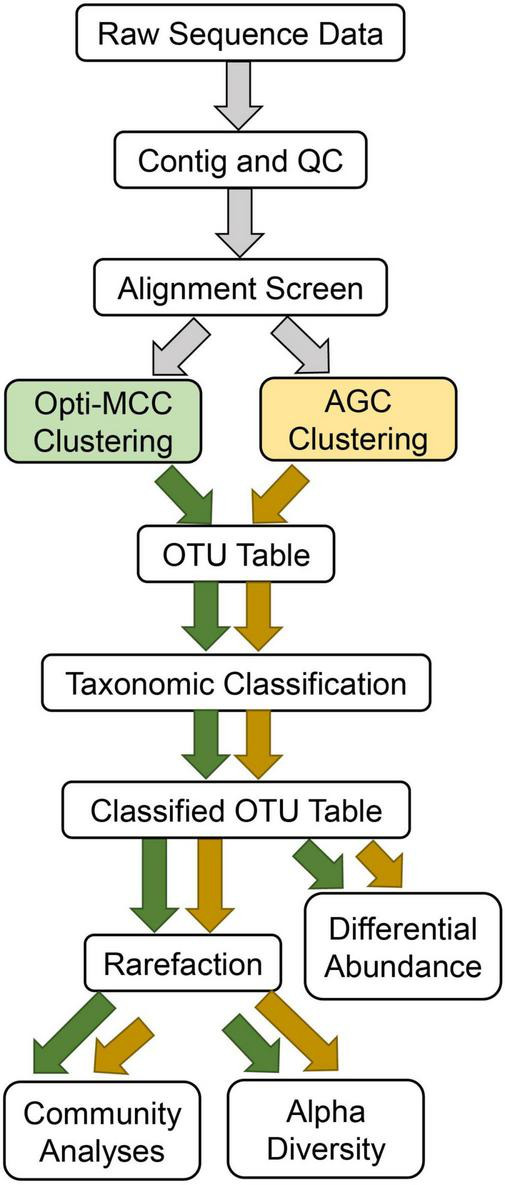
Workflow used for each analysis.

Amplicon sequences were processed in Mothur using pipelines which differed only in the clustering method used (Figure 1). Briefly, contigs were first created using make.contigs, which compares agreement between the overlapping portion of reads to identify low confidence bases. *nifH* contig average length was 362 bp, with an average overlapping region of 137 bp. *nrfA* contigs averaged 240 bp and had an average overlapping region of 162 bp. Contigs were then screened using screen.seqs to remove any sequences with ambiguous bases identified by mismatches during contig-building. Next, sequences were aligned to reference alignments using align.seqs. *nifH* sequences were aligned to the FunGene sequence database for *nifH* and *nrfA* sequences were aligned to a reference database generated during an earlier, comprehensive shotgun sequencing effort including soils from the location sampled for this experiment (Orellana et al., 2018). The aligned sequences were then screened again using screen.seqs followed by filter.seqs to remove sequences not aligned within the expected region based on start and end position, and to discard any sequences containing 8 or more homopolymers. Remaining unique, quality-filtered sequences were then clustered using pre.cluster followed by dist.seqs using either the AGC clustering approach implemented with VSEARCH, or the Opti-MCC method, both at a 97% similarity cutoff. These approaches will be referred to as AGC-0.03 and MCC-0.03 hereafter. Because the AGC method routinely produces fewer OTUs than the Opti-MCC method, an additional trial of AGC was included at 98% similarity (AGC-0.02) to evaluate whether differing results between AGC and Opti-MCC were the result of OTU counts. Coverage was calculated for each sample in Mothur using rarefaction.single followed by summary.single commands with the inverse Simpson metric. Representative sequences for each OTU were taxonomically classified using the Wang method (Wang et al., 2007) implemented in Mothur. For *nifH*, the FunGene database was used for classification; for *nrfA* we present results for both the FunGene database as well as a novel clade-based taxonomic database. The latter was created by prepending the clade designations identified in Welsh et al. (2014) to each taxonomic rank to facilitate taxonomic classification for genes like *nrfA* whose functional gene and phylogenetic markers have incongruent evolutionary histories. This allows for better differentiation between taxonomic groups which appear within more than one clade of *nrfA*. Cutoffs for taxonomic classification were set at 70% similarity for *nifH* and 50% similarity for *nrfA*, based on estimates generated in ClustalW for average percent identity between the sequences in the FunGene database for each (71.68 and 50.34% similarity, respectively). Amplicon sequence data for 16S rRNA genes and N-cycling functional genes are available for download on the NCBI SRA database *via* the BioProject accession number: PRJNA752786.^1^

### Analyses and Metrics Assessed

The resulting OTU tables were subjected to identical downstream analyses (Figure 1). For analyses requiring even read depth, ten independent rarefactions were executed using command rarefy_even_depth in R package Phyloseq. OTU tables were rarefied to the lowest read depth present. Each OTU table was rarefied using the same array of ten random seeds to ensure comparability and repeatability. Skewness of unrarefied OTU tables was assessed using Fisher’s Skewness implemented in R package MultiSkew, and homogeneity of dispersions was assessed using PERMDISP implemented in R package vegan.

Alpha diversity was estimated using the Chao1 richness index and Shannon diversity index. The Chao1 index emphasizes rare organisms and predicts the number of taxa in a sample by extrapolating rare taxa that may have been missed due to undersampling. The Shannon index combines both richness and evenness to quantify the uncertainty associated with predicting a randomly sample taxon. By comparing these two indices between methods, we can determine whether the OTU tables generated by each differ in richness or evenness. Chao1 and Shannon estimates were generated for each OTU table using estimate_richness function in Phyloseq, corrected for multiple comparisons using Tukey’s HSD at α = 0.05. Conclusions regarding which treatment groups were more or less diverse than others were compared between pipeline datasets to determine if richness comparisons between treatment groups are biased by upstream clustering. Subtle differences in the information provided by each metric may be used to identify the mechanism by which pipelines produce different qualitative conclusions downstream, if any.

Multivariate analyses PERMANOVA and ANOSIM were used to determine community-level differences among treatments, using the adonis and anosim functions in R package vegan (Oksanen et al., 2019), corrected for multiple comparisons using the Benjamini-Hochberg method (Benjamini and Hochberg, 1995), the multivariate analog of Tukey’s HSD. The number of trials out of ten independent rarefaction trials for which each pairwise comparison was significant at α = 0.05 were tallied and compared between pipelines. Both agreement between each clustering methods’ individual pairwise conclusions (i.e., communities significantly different or not) as well as robustness to repeated rarefaction (i.e., number of times among ten rarefaction trials that treatment differences were found to be significant) were considered. PERMANOVA is a permutational, non-parametric analog of the MANOVA, a centroid-based analysis of variance for multivariate datasets. Therefore, null hypotheses rejected during PERMANOVA analyses denote a significant difference between multivariate centroids specifically. ANOSIM, however, is a rank-based omnibus test which is sensitive to differences in centroid, as well as other underlying aspects of data structure, including skewness, correlation, and more. Differences in conclusions between the two analyses therefore shed light on which aspects of the underlying data differ between agricultural treatment groups.

OTUs that were differentially abundant between one or more treatment groups were identified for each resulting pipeline dataset using the parametric Wald test in R package DESeq2. As this package implements a negative binomial mixed model designed to circumvent the need for rarefaction, this analysis was applied only to unrarefied datasets. Beta diversity comparisons are generally unaffected by extremely rare taxa; therefore, only the top 100 most abundant OTUs were considered for this analysis. Agreement between pipelines was assessed based on agricultural treatments found to have higher or lower abundance of identified taxa.

## Results

### Operational Taxonomic Unit Characteristics*—nifH*

As anticipated, the AGC-0.03 method produced the fewest OTUs for a total of 4,242 (Table 1). The next largest OTU count was produced by the MCC-0.03 method with 5,810 OTUs generated. Tightening the similarity threshold for the AGC method to 98% resulted in roughly twice as many OTUs as the other methods. Among methods, singleton OTUs represented comparable proportions of total OTUs (Table 1). At a 97% similarity cutoff, the MCC-0.03 method yielded 28% more non-singleton OTUs and 43% more singleton OTUs than the AGC-0.03 method.

**TABLE 1:** *nifH* OTU table and rarefaction characteristics for each clustering method.

Method	OTUs pre-rarefaction	Singleton OTUs	Percent singletons	Avg OTUs post-rarefaction	OTUs lost post-rarefaction	Percent lost
AGC-0.03	4,242	2,768	65%	444	3,798	90%
MCC-0.03	5,810	3,928	68%	583	5,227	90%
AGC-0.02	9,768	7,033	72%	687	9,081	93%

The lowest read depth for the *nifH* dataset was 182, which represented an average coverage ranging from 90.7 to 97.1% for the AGC-0.03 method, and a range from 87.7 to 95.7% for the MCC-0.03 method (Table 2). Rarefaction to this depth resulted in 90% fewer OTUs for both AGC-0.03 and MCC-0.03 methods, and 93% for the AGC-0.02 method. Skewness within the unrarefied OTU tables followed similar patterns for both the AGC and MCC methods, with the AGC-0.03 method producing slightly less skewness. This is largely the result of the higher number of singleton OTUs generated using the MCC method. Despite this, dispersions for each treatment were homogenous for both methods, averaging approximately 0.65 distance to median for all treatments and methods.

**TABLE 2:** *nifH* OTU table statistics for each agricultural treatment.

Method	Treatment	Avg rarefied coverage	Unrarefied skewness	Avg unrarefied distance to median
AGC-0.03	T1	96.7%	37.9	0.654
	T2	95.8%	29.4	0.653
	T3	97.1%	42.9	0.653
	T4	90.7%	28.2	0.654

MCC-0.03	T1	95.2%	42.5	0.655
	T2	94.9%	32.1	0.652
	T3	95.7%	45.5	0.654
	T4	87.7%	31.8	0.643

### Alpha Diversity*—nifH*

**FIGURE 2:**
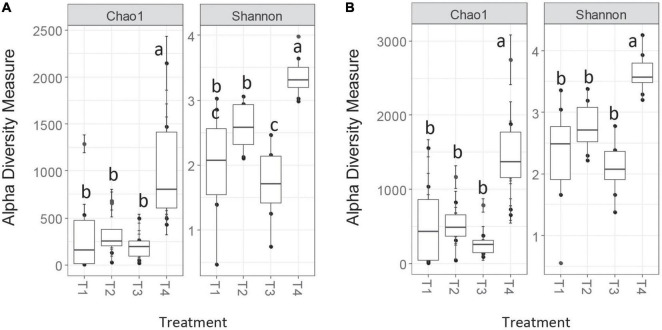
Chao1 and Shannon alpha diversity measures for *nifH* for AGC-0.03 **(A)** and MCC-0.03 **(B)** methods. Letters indicate significance at α < 0.05 *via* ANOVA with Tukey’s HSD correction for multiple comparisons.

Alpha diversity measures on the unrarefied OTU tables were very similar between the two methods, with Chao1 in good agreement and Shannon exhibiting similar patterns with slightly differing pairwise comparison differences (Figure 2). Chao1 richness estimates were slightly larger in magnitude for the MCC method compared to AGC. These similarities between methods persisted post-rarefaction, and results did not differ between rarefaction trials.

### Community Analyses*—nifH*

Multivariate community analyses on rarefied datasets agreed among all methods. All pairwise comparisons except T1 vs. T3 generated significant ANOSIM results, with *R*-values ranging from 0.2 to 0.9. For each pairwise comparison, *R*-values differed only slightly between methods, and would be considered qualitatively the same in terms of describing relative effect sizes.

For all pairwise comparisons except T3 vs. T4, PERMANOVA results were always in agreement among independent rarefaction trials, at either 10/10 rarefied datasets yielding significant community differences, or 0/10 (Table 3). These results remained consistent when considering only the top 100 OTUs, which represented 83 and 76% of the total sequences for the AGC-0.03 and MCC-0.03 datasets, respectively. In the case of the T3 vs. T4 comparison, neither method generated consistent PERMANOVA results across rarefaction trials. This was despite consistently significant ANOSIM results, and comparatively the highest effect size according to ANOSIM, at *R* ≈ 0.9. These two agricultural treatments represent the most skewed and least skewed OTU data for both methods, suggesting that ANOSIM can detect this difference in skewness between treatments.

**TABLE 3:** *nifH* community analysis results for each clustering method across ten independent rarefaction trials.

Treatment comparison	Method	Avg sig ANOSIM rarefied R	Sig PERMANOVA of 10 rarefactions for all OTUs	Sig PERMANOVA of 10 rarefactions for top 100 OTUs
T1 vs. T2	AGC-0.03	0.282	10	10
	MCC-0.03	0.360	10	10
	AGC-0.02	0.309	10	10

T1 vs. T3	AGC-0.03	n.s.	10	10
	MCC-0.03	n.s.	10	10
	AGC-0.02	n.s.	10	10

T1 vs. T4	AGC-0.03	0.640	0	0
	MCC-0.03	0.671	0	0
	AGC-0.02	0.624	0	0

T2 vs. T3	AGC-0.03	0.297	0	0
	MCC-0.03	0.287	0	0
	AGC-0.02	0.315	0	0

T2 vs. T4	AGC-0.03	0.717	10	10
	MCC-0.03	0.741	10	10
	AGC-0.02	0.775	10	10

T3 vs. T4	AGC-0.03	0.797	2	7
	MCC-0.03	0.876	4	9
	AGC-0.02	0.839	2	6

*Significance assessed at α = 0.05. n.s. in various spots means “not significant.”*

The variance explained by each PERMANOVA model, represented as its *R*^2^ value, compared to its adjusted *p*-value followed very similar trends for all methods (Supplementary Figure 1). The range of effect sizes identified in all OTUs was lower for the AGC-0.02 method, due to the presence of twice as many OTUs as the others. For the top 100 OTUs, the MCC-0.03 method yielded a higher upper end to the range of effect sizes.

### Differential Abundance*—nifH*

**FIGURE 3:**
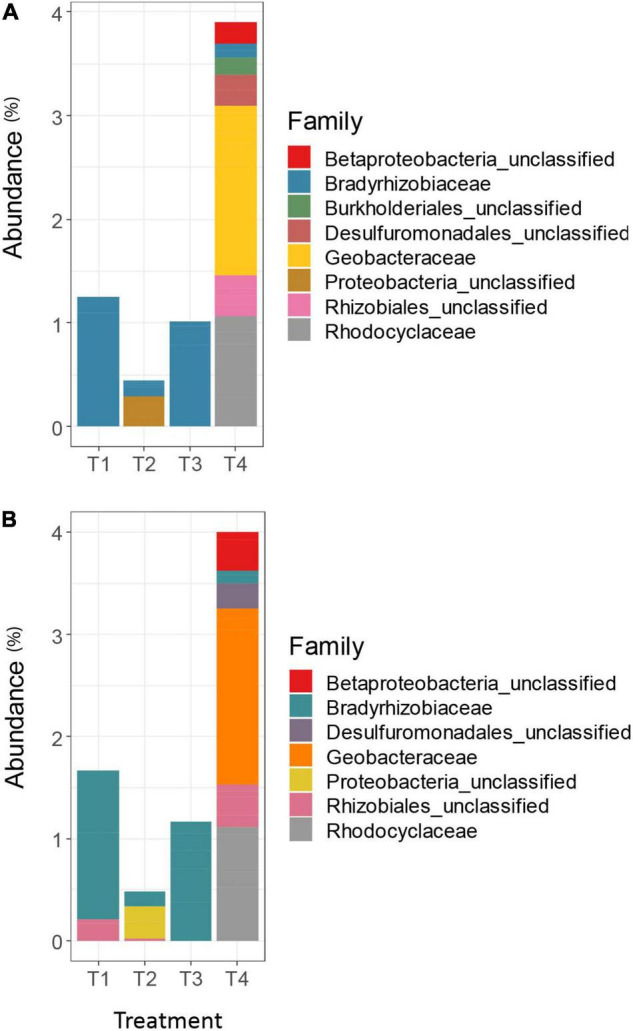
Differentially abundant *nifH* taxa among the top 100 OTUs for the AGC-0.03 method **(A)** and the MCC-0.03 method **(B)**. Significance of differential abundance assessed at α = 0.01.

Differential abundance analysis using DESeq2 on the unrarefied OTU tables generated results that were largely in agreement (Figure 3). The MCC-0.03 method detected higher relative abundance of *Rhizobiales* in both T1 and T4 treatments, compared to the other treatments, but AGC-0.03 only detected this taxon in higher relative abundance in the T4 treatment.

### Operational Taxonomic Unit Characteristics*—nrfA*

Similar to the trends seen in the *nifH* datasets, the AGC-0.03 method produced the fewest OTUs, followed by MCC-0.03 and then AGC-0.02 (Table 4). The AGC-0.03 method generated 12,144 OTUs, of which 5,280 (43%) were singleton OTUs, compared to 16,497 OTUs and 8,320 (50%) singletons generated by the Opti-MCC method at the same cutoff. In contrast to results for *nifH*, the AGC-0.02 method applied to *nrfA* produced comparatively more OTUs than the other methods, generating nearly 4 times more OTUs compared to AGC-0.03 and nearly 3 times more OTUs over MCC-0.03.

**TABLE 4:** *nrfA* OTU table and rarefaction characteristics for each clustering method.

Method	OTUs pre-rarefaction	Singleton OTUs	Percent singletons	Avg OTUs post-rarefaction	OTUs lost post-rarefaction	Percent lost
AGC-0.03	12,144	5,280	43%	7,791	4,353	36%
MCC-0.03	16,497	8,320	50%	9,912	6,585	40%
AGC-0.02	44,495	24,323	55%	25,367	19,128	43%

The lowest read depth for the *nrfA* dataset was 4,096, which resulted in average coverages ranging from 93.4 to 96.2% for AGC-0.03 and 92.3–94.6% for MCC-0.03 (Table 5). Rarefying to this depth resulted in an average of 7,791 OTUs, or a loss of 36% for the AGC-0.03 method, and an average of 9,912 OTUs, or a loss of 40%, for MCC-0.03. In all cases, the percent lost upon rarefaction was comparatively smaller than it was for *nifH*. The reduction in loss upon rarefaction was partly due to the skewness for the *nrfA* datasets, which was much higher than for *nifH*, ranging from 40.4 up to 64.6. This indicates that a larger proportion of the data are “smeared” out toward the tail, and thus random subsampling is more likely to collect data points from the full range, compared to a dataset which is less skewed. Dispersion for *nrfA* was homogeneous between all agricultural treatments and clustering methods, and ranged narrowly from 0.644 to 0.652 distance to sample median.

**TABLE 5:** *nrfA* OTU table statistics for each agricultural treatment.

Method	Treatment	Avg rarefied coverage	Unrarefied skewness	Avg unrarefied distance to median
AGC-0.03	T1	95.2%	59.0	0.645
	T2	93.4%	41.5	0.644
	T3	96.2%	61.5	0.648
	T4	95.3%	40.4	0.648

MCC-0.03	T1	93.7%	60.8	0.646
	T2	92.3%	24.6	0.649
	T3	94.6%	64.6	0.651
	T4	93.8%	42.5	0.652

### Alpha Diversity*—nrfA*

**FIGURE 4:**
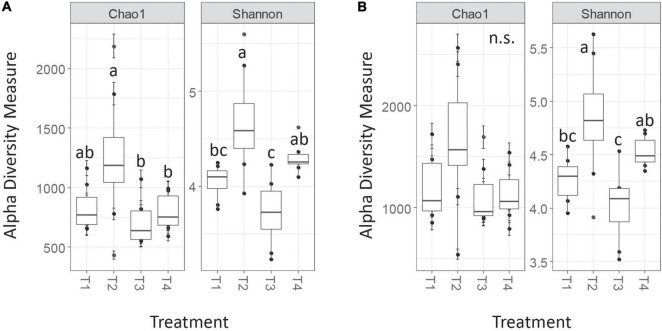
Chao1 and Shannon alpha diversity measures for *nrfA* for AGC-0.03 **(A)** and MCC-0.03 **(B)** methods. Letters indicate significance at α < 0.05 *via* ANOVA with Tukey’s HSD correction for multiple comparisons.

Trends in *nrfA* alpha diversity metrics were roughly similar between the AGC and MCC clustering approaches, though statistical significance of pairwise comparisons for certain metrics differed in some cases (Figure 4). The MCC-0.03 method detected no significant differences in Chao1 richness among treatments, while several pairwise treatment differences occurred in the OTU table generated *via* the AGC-0.03 method. Additionally, the magnitude of these estimates varied, with Chao1 estimates from the AGC-0.03 OTU table being slightly higher than that of the MCC-0.03, opposite of the trend observed for *nifH*. Shannon diversity estimates, which consider evenness in addition to richness, did not differ between the AGC and MCC methods. Similar to *nifH*, these trends for *nrfA* alpha diversity persisted post-rarefaction and did not differ between rarefaction trials.

### Community Analyses*—nrfA*

ANOSIM results for all pairwise agricultural treatment comparisons were significant across all clustering methods, with effect sizes (R) ranging from 0.492 to 0.759 (Table 6). Effect sizes for each pairwise comparison were similar between MCC-0.03 and AGC-0.03 methods, with AGC-0.02 exhibiting slightly lower *R*-values due to its substantially larger OTU count. Overall, agricultural treatment effect sizes were similar among clustering methods, yielding the same qualitative interpretations.

**TABLE 6:** *nrfA* community analysis results for each clustering method across 10 independent rarefaction trials.

Treatment comparison	Method	Avg sig ANOSIM rarefied R	Sig PERMANOVA of 10 rarefactions for all OTUs	Sig PERMANOVA of 10 rarefactions for top 100 OTUs
T1 vs. T2	AGC-0.03	0.590	4	10
	MCC-0.03	0.583	10	0
	AGC-0.02	0.492	7	0

T1 vs. T3	AGC-0.03	0.590	2	9
	MCC-0.03	0.584	10	7
	AGC-0.02	0.559	1	0

T1 vs. T4	AGC-0.03	0.714	0	0
	MCC-0.03	0.731	0	0
	AGC-0.02	0.618	0	0

T2 vs. T3	AGC-0.03	0.729	4	1
	MCC-0.03	0.715	10	0
	AGC-0.02	0.624	9	0

T2 vs. T4	AGC-0.03	0.759	10	10
	MCC-0.03	0.724	10	0
	AGC-0.02	0.685	10	0

T3 vs. T4	AGC-0.03	0.738	0	0
	MCC-0.03	0.670	0	0
	AGC-0.02	0.607	0	0

*Significance assessed at α = 0.05.*

PERMANOVA results differed substantially between clustering methods, with only the MCC method yielding consistent qualitative results among rarefaction trials at either 10/10 or 0/10 significant results obtained for each pairwise agricultural treatment comparison. For three of the four pairwise comparisons that the MCC method identified as being significant in all rarefaction trials, the AGC method only returned significant treatment differences in 20–40% of the trials. The fourth pairwise comparison was the only one for which the AGC method produced significant results in all rarefaction trials. For the two pairwise comparisons in which the MCC method yielded no significant treatment differences in any rarefaction trial, the AGC method also yielded no significant results from any rarefaction trial.

For analyses of only the top 100 OTUs, which represented 45 and 42% of total reads for AGC-0.03 and MCC-0.03 respectively, results also differed depending on clustering method. The MCC-0.03 approach did not produce significant pairwise agricultural treatment differences in the top 100 OTU communities except for one pairwise comparison (T1 vs. T3), in which it produced a significant treatment difference in 7 out of 10 rarefaction trials. Conversely, the AGC method tended to identify significant treatment differences more consistently among the top 100 OTUs compared to all OTUs. For example, for T1 vs. T2, the AGC method produced significant differences in all ten rarefaction trials when evaluating the top 100 OTUs only, as opposed to only four of the trials when evaluating all OTUs. These results indicate that the AGC-0.03 method partitions variation due to treatment differences into the most abundant (top) OTUs.

Increasing the OTU number by increasing the similarity cutoff for the AGC method to 98% tended to increase the consistency of results among rarefaction trials using all OTUs, though still not to the 100% consistency achieved by the MCC method. In addition, the AGC-0.02 method produced no agricultural treatment differences among the communities comprised of the top 100 OTUs, as these abundant OTUs represented a small percentage of the total OTUs in the dataset.

The pairwise treatment comparisons which did not yield consistent PERMANOVA results on AGC-clustered datasets were generally those with comparatively lower *R*-values *via* ANOSIM, implying a smaller effect size for those comparisons. A comparison of the *R*^2^ values of the PERMANOVA trials vs. their adjusted *p*-values shows the MCC-0.03 approach produces OTU tables which yield significant *p*-values at lower effect sizes than the AGC-0.03 approach (Supplementary Figure 2). Conversely, while the AGC-0.02 method is able to produce significant results at similarly low effect sizes, proportionally fewer of the treatment comparisons are found to be significantly different. When considering only the top 100 OTUs, the MCC-0.03 method was able to produce effect sizes comparable to the AGC-0.03 method, but far fewer of these achieved a significant *p*-value compared to the AGC-0.03 method. In contrast to *nifH*, the clustering methods applied to *nrfA* led to greater differences in effect size and detectable significant treatment differences in downstream analyses of all OTUs.

### Differential Abundance*—nrfA*

**FIGURE 5:**
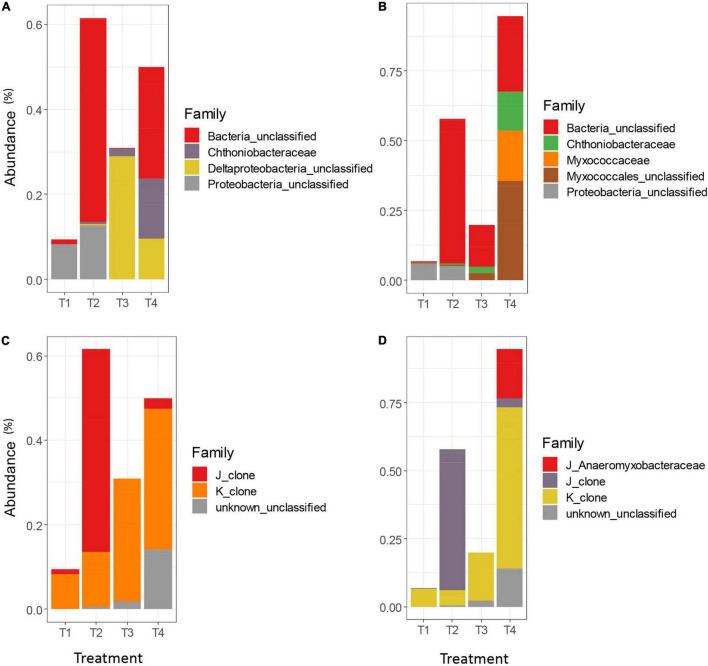
Differentially abundant *nrfA* taxa among the top 100 OTUs for the AGC-0.03 method classified using the FunGene database **(A)**, the MCC-0.03 method using the FunGene database **(B)**, the AGC-0.03 method using the clade-based database **(C)**, and MCC-0.03 using the clade-based database **(D)**. Significance of differential abundance assessed at α = 0.01.

While the overall trends in differential abundance among agricultural treatments were generally similar between clustering methods, there were some marked differences between the AGC and MCC approaches, and between taxonomic databases. The FunGene database often failed to classify representative sequences for many differentially abundant OTUs beyond the Kingdom level, resulting in a much larger proportion of unclassified Bacteria in the FunGene analysis (Figures 5A,B) compared to the clade-based taxonomic database (Figures 5C,D). Within the FunGene-classified OTUs for AGC and MCC, classifications for differentially abundant taxa were generally similar. However, several differentially abundant Myxococcales OTUs were identified from the MCC OTU table, whereas similar sequences were classified less granularly as Deltaproteobacteria in the AGC OTU table. The relative abundances of these OTUs also differed across treatments, with these taxa appearing more abundant in the T3 treatment in the AGC OTU table, but appearing more abundant in the T4 treatment in the MCC OTU table. Both methods identified Chthoniobacteraceae, the only non-Proteobacteria phyla identified, as more abundant in the T4 treatment, as well as the T3 treatment.

The clade-based taxonomic database uses a clade designation prefix to aid the classification algorithm in differentiating between taxa whose organismal phylogeny is mixed between clades. Using this database, the AGC and MCC OTU tables followed differential abundance trends similar to those found when using the FunGene database (Figures 5C,D). As with the FunGene-classified database, the MCC method yielded a slightly longer list of differentially abundant taxa, with the organisms missing from the AGC method appearing primarily in the T4 treatment. Both methods resulted in a small number of unclassified differentially abundant OTUs in the T3 treatment and a slightly larger number in the T4 treatment. Both methods also were generally in agreement regarding the relative abundances of OTUs which resembled clones from clades J and K. However, only the MCC method allowed for identification of Anaeromyxobacteraceae belonging to clade J. Because the AGC-0.02 method produced so many OTUs, the top 100 OTUs only contained two OTUs which were differentially abundant among agricultural treatments, both of which were classified as Clade J for both methods (data not shown).

## Discussion

The processing necessary to convert raw Illumina amplicon sequence data to a usable OTU table can be achieved through any number of approaches within the continuously growing toolbox of bioinformatics methods. However, the choices made at each step during upstream processing are liable to impact downstream results, and the ways these choices influence different types of data are only beginning to be explored. In this study, we conducted a comparison of OTU tables generated from pipelines which differed only in their clustering method on each of two functional genes: *nifH*, the diagnostic gene for N-fixation, which is comprised of only 3 clades, and *nrfA*, the diagnostic gene for DNRA, which is comprised of at least 18 clades. AGC, a quicker heuristic method, performed comparably to the slower, more accurate hierarchical Opti-MCC method for the phylogenetically narrow gene, *nifH*. For *nrfA*, however, which harbors a much greater diversity than does *nifH*, the AGC method did not perform as well as the Opti-MCC method, producing an OTU table which resulted in unreliable downstream community analyses post-rarefaction and evidence of reduced granularity in classifying OTU taxonomy. Our results demonstrate how clustering methods optimized for 16S rRNA phylogenies may perform differently depending on the diversity and lineages of the functional gene sequences being processed.

Many of the differences observed between results from the two methods can be attributed to the strategy each uses to assign sequences to clusters. The Opti-MCC method initializes each unique sequence as its own OTU, and proceeds to combine these based on all-against-all similarity comparisons for the sequences in each cluster (Westcott and Schloss, 2017). The metric used to optimize cluster assignment, the MCC, represents a balance of not only true positives, but also false positives, true negatives, and false negatives. In contrast, the AGC method approximates this process by assigning sequences to clusters based on similarity to an averaged centroid sequence, beginning with the most abundant OTUs (He et al., 2015). As a “greedy” algorithm, it places a sequence with the first OTU it finds whose centroid sequence is within the chosen similarity cutoff. Since these comparisons begin by considering the most abundant OTUs, there is a risk of placing a sequence with an abundant OTU when it would be more accurately classified in a smaller OTU which was not considered for comparison. In other words, OTU tables generated using AGC may have a higher rate of false positives within the most abundant OTUs, and a higher rate of false negatives in rarer OTUs. This is manifested in the differing performance of the AGC method on genes of differing diversity, as the full breadth of diversity is more likely to be represented among the most abundant OTUs for a phylogenetically narrow gene compared to a broad one.

The clearest demonstration of this bias toward the most abundant OTUs in AGC can be seen in the differing level of consistency among PERMANOVA results for the full OTU table vs. only the 100 most abundant OTUs. While both AGC and Opti-MCC methods produced consistent PERMANOVA results for analyses of all OTUs and only the top 100 OTUs for *nifH*, they differed in performance for *nrfA*. The Opti-MCC method yielded reliable results using the full OTU table, but many pairwise differences became undetectable when analyzing only the top 100 OTUs. This indicates that there is enough explanatory power in the data beyond of the top 100 OTUs that differences become difficult to detect when the less abundant OTUs are excluded. For the AGC method, however, we observed the opposite: for many PERMANOVA results which were inconsistent among rarefaction trials when the full dataset was analyzed, results became more reliable when only the top 100 OTUs were considered. These results demonstrate that explanatory power is concentrated more heavily in the most abundant OTUs when clusters are assembled using AGC.

Although the Opti-MCC method failed to produce consistent PERMANOVA results using only the top 100 OTUs, the taxonomic classification within the top 100 OTUs was more granular than that achieved from AGC. This speaks to the accuracy of the original cluster assignment, as fewer false positives result in a more precise representative sequence which can be better resolved against a reference database. When the AGC method assigns sequences preferentially to abundant OTUs without searching for a better fit among less abundant OTUs, the result is “fuzzier” clusters with a wider variety of sequences, resulting in an increasingly generic representative sequence. This may lead to poorer granularity in taxonomic classification. While we can circumvent some of the drawbacks of AGC-clustered OTUs by focusing only on the most abundant OTUs, taxonomy assignments may still suffer.

In contrast to the variable results obtained using PERMANOVA, the results from the same datasets were strikingly consistent when analyzed using ANOSIM: across all clustering methods and rarefaction trials, ANOSIM results consistently agreed in both statistical significance and approximate effect size. ANOSIM is a rank-based omnibus test which detects differences in several aspects of underlying data structure (Anderson and Walsh, 2013). Therefore, the observed consistency in results among clustering methods indicates that the agricultural treatment differences present in the sequence data were preserved in both the AGC and Opti-MCC datasets—and still differ with the same quantifiable magnitude—but that these differences are simply being structured differently within the OTU tables. In the case of the AGC-constructed *nrfA* OTUs, this structure made it difficult to detect these treatment differences *via* PERMANOVA when assessing the full OTU table. In addition to consistent ANOSIM results, processed data from both methods shared many similarities in terms of alpha diversity metrics. Results generated from each method followed the same treatment patterns and exhibited comparable effect sizes, for both the unrarefied OTU tables as well as each independently rarefied OTU table. While the outcomes of some analyses may differ between methods, these results demonstrate that the two can still produce very similar results for others.

While tightening the similarity cutoff for the AGC method resulted in many more OTUs, it still did not produce the same consistency among rarefied analytical results as the Opti-MCC method. While some pairwise comparisons improved in reliability, e.g., two trials resulting in 4/10 significant differences detected at 97% similarity improved to 7/10 and 9/10 significant under 98% similarity, others did not improve. In addition, the large number of OTUs rendered detection of differences within the top 100 OTUs impossible, and consideration of only the top 100 OTUs was no longer appropriate for determining differential abundance among treatments. Therefore, although using a higher similarity threshold marginally improved performance, it introduced additional issues, such as the need to reevaluate and identify appropriate cutoffs for differential abundance analyses. This clearly demonstrates the importance of prioritizing quality of OTU cluster formation over quantity of OTUs, as more is not necessarily better.

This study aimed to explicitly compare the impact of two OTU clustering algorithms on downstream analyses while holding constant all other aspects of the bioinformatics pipeline. While this approach allowed us to directly quantify the impact of clustering algorithm alone, this also limits the comparison to clustering approaches that may be implemented using the same pipeline and otherwise identical steps. However, an increasing number of researchers are beginning to turn to the use of amplicon sequence variants (ASVs) in lieu of OTUs, an approach which “denoises” the unprocessed sequence reads by clustering them into biologically meaningful groups independently of a predefined level of similarity (Hugerth and Andersson, 2017). A popular implementation of this approach is through the package DADA2 (Callahan et al., 2016), which begins by initializing clusters based on amplicon abundance (where, as in the AGC heuristic, more common sequences are assumed to be more biologically accurate) and sequence distance from other reads. The quality scores assigned to each base by the sequencing platform are then used to build an error model to “correct” reads by assigning low frequency reads to higher frequency reads from which they may have been derived *via* sequencing errors (Hugerth and Andersson, 2017). While this approach circumvents some of the pitfalls of OTU clustering at a fixed threshold, it is important to note that such denoising algorithms are still performing clustering operations and many of the same considerations regarding how performance may vary between functional genes of contrasting diversity may still apply.

Functional genes are increasingly assessed in microbiome research because they can shed light on the ways that our experimental treatments impact different functional group communities and the ecological processes they perform. However, in some cases, these functional genes represent a vast range of diversity and divergent lineages, with some comprised of only a few clades, and others an order of magnitude more. This study demonstrated some of the differences that can manifest when applying differing clustering algorithms to sequences from genes on opposite ends of the diversity spectrum. While the AGC method performed well on a gene with little diversity, it resulted in unreliable analytical outcomes for some tests when applied to a more diverse gene. This is especially problematic when we consider that functional gene community analyses are typically presented together as a means to demonstrate which functional groups are differentially impacted by a treatment. If we choose a method which obstructs detection of treatment differences in diverse genes, we introduce biased Type II errors by failing to identify treatment effects in some genes but not in others. Surprisingly, despite rarefying to a very low read depth for *nifH*, Type II errors were not introduced, and treatment differences were detected consistently among methods. This emphasizes the importance of considering gene characteristics when selecting methods, as more Type II errors were introduced simply by clustering *nrfA* sequences with the AGC method than were introduced by rarefying to a low read depth for *nifH*.

Ultimately, the reliability of our downstream analyses and subsequent conclusions is only as good as our upstream processing. For questions which can be answered using tests agnostic to clustering method (e.g., ANOSIM), or for genes of relatively low phylogenetic diversity (e.g., *nifH*), most upstream processing methods should lead to similar conclusions from downstream analyses. For studies involving more diverse genes, however, care should be exercised to ensure accurate clustering for all genes. Most importantly, we must continually reassess the performance of our preferred bioinformatics tools as technology continues advancing and more sophisticated methods become available.

## Data Availability Statement

Amplicon sequence data for 16S rRNA genes and N-cycling functional genes are available for download on the NCBI SRA database *via* the BioProject accession number: PRJNA752786 (https://www.ncbi.nlm.nih.gov/sra/PRJNA752786).

## Author Contributions

SE conceived of the study, conducted the study, and wrote the manuscript. RS, AK, and WY provided guidance and edited the manuscript. All authors contributed to the article and approved the submitted version.

## Conflict of Interest

The authors declare that the research was conducted in the absence of any commercial or financial relationships that could be construed as a potential conflict of interest.

## Publisher’s Note

All claims expressed in this article are solely those of the authors and do not necessarily represent those of their affiliated organizations, or those of the publisher, the editors and the reviewers. Any product that may be evaluated in this article, or claim that may be made by its manufacturer, is not guaranteed or endorsed by the publisher.

## Footnotes

1
https://www.ncbi.nlm.nih.gov/sra/PRJNA752786
