# Supplementary material for: Nitrogen Cycling Microbial Diversity and Operational Taxonomic Unit Clustering: When to Prioritize Accuracy Over Speed
Source: Front Microbiol. 2022 May 26;13:730340. doi: 10.3389/fmicb.2022.730340 (PMC9201982; doi:10.3389/fmicb.2022.730340)
Supplement: Supplementary file 1 [file Data_Sheet_1.pdf]

Nitrogen cycling microbial diversity and OTU clustering: when to prioritize accuracy over speed

Sada Egenriether[1], Robert Sanford[2], Wendy H. Yang[1,2,3], Angela D. Kent[1,4*]

[1]Program in Ecology, Evolution and Conservation Biology, University of Illinois at Urbana-Champaign, Urbana, IL, USA
[2]Department of Geology, University of Illinois at Urbana-Champaign, Urbana, IL, USA
[3]Department of Plant Biology, University of Illinois at Urbana-Champaign, Urbana, IL, USA
[4]Department of Natural Resources and Environmental Sciences, University of Illinois at Urbana-Champaign, Urbana, IL, USA.

*** Correspondence:**
Angela D. Kent
akent@illinois.edu

Supplemental material

Table S1. *nrfA* and *nifH* gene primers used for Illumina sequencing.

Target gene	Primers	Primer sequence (5'→3')	Reference
*nrf*A	nrfAF2awMOD	GSI CAR TGY CAY GTI GAR TA	Cannon *et al.* 2019
	nrfAR1MOD	GGC ATR TGR CAR TCI RYR CA	
	nrfAF2awMODgeo	GSI CAR TGY CAY GTI ASB TA	
*nif*H	PolF	TGCGAYCCSAARGCBGACTC	Poly *et al.* 2001
	PolR	ATSGCCATCATYTCRCCGGA	

Table S1. Starting contig count and post-processing read count for *nrfA* and *nifH*.

Treatment	Replicate Sample	*nifH* Contigs	*nrfA* Contigs	*nifH* Reads Postprocessing	*nrfA* Reads Postprocessing
T1	1	756	13,606	182	12,777
	2	851	10,159	415	9,114
	3	823	13,377	449	12,106
	4	4,059	17,243	3,006	15,926
	5	17,657	5,524	14,509	5,054
	6	14,647	9,769	10,921	9,095
	7	12,266	11,654	10,342	10,705
	8	7,110	6,703	5,620	6,345
T2	1	16,857	16,516	13,737	15,705
	2	13,132	15,358	10,057	14,751
	3	12,439	4,286	11,321	4,096
	4	15,398	9,526	10,657	8,864
	5	6,302	16,624	5,335	15,775
	6	981	12,925	730	12,091
	7	6,331	12,079	5,193	11,526
	8	6,470	12,055	4,756	11,291
T3	1	6,290	20,996	4,810	19,400
	2	6,802	8,188	5,632	7,776
	3	6,202	14,596	4,599	13,464
	4	1,804	12,204	1,323	11,415
	5	15,128	17,690	9,248	16,635
	6	7,171	8,681	4,377	7,427
	7	5,638	7,393	3,575	6,897
	8	13,192	9,313	7,362	8,349
T4	1	13,806	8,289	12,418	7,687
	2	9,320	7,677	7,740	7,253
	3	13,452	6,076	11,849	5,641
	4	16,677	11,262	13,598	10,673
	5	10,442	8,999	8,880	8,330
	6	7,801	12,043	6,131	11,276
	7	5,217	9,203	4,136	8,506
	8	5,117	15,089	4,446	13,832

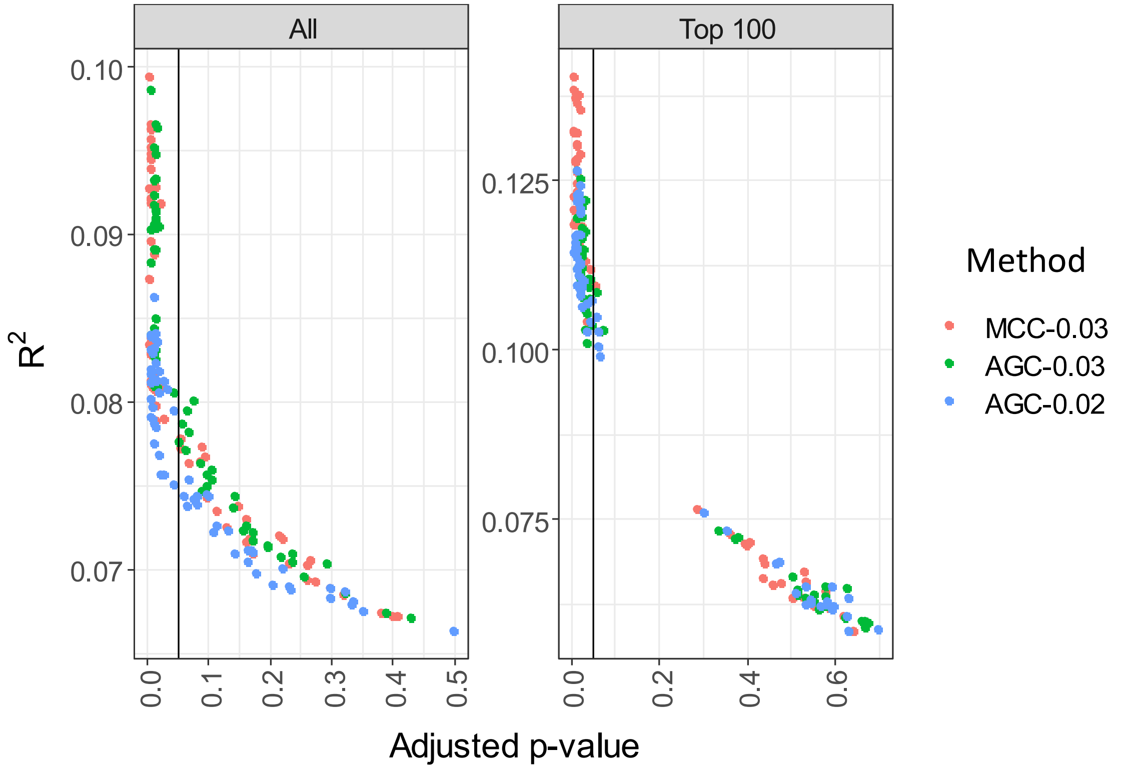

Figure S1. Effect size vs significance for all PERMANOVA trials on independently rarefied datasets for *nifH*. Each point represents 1 PERMANOVA model.

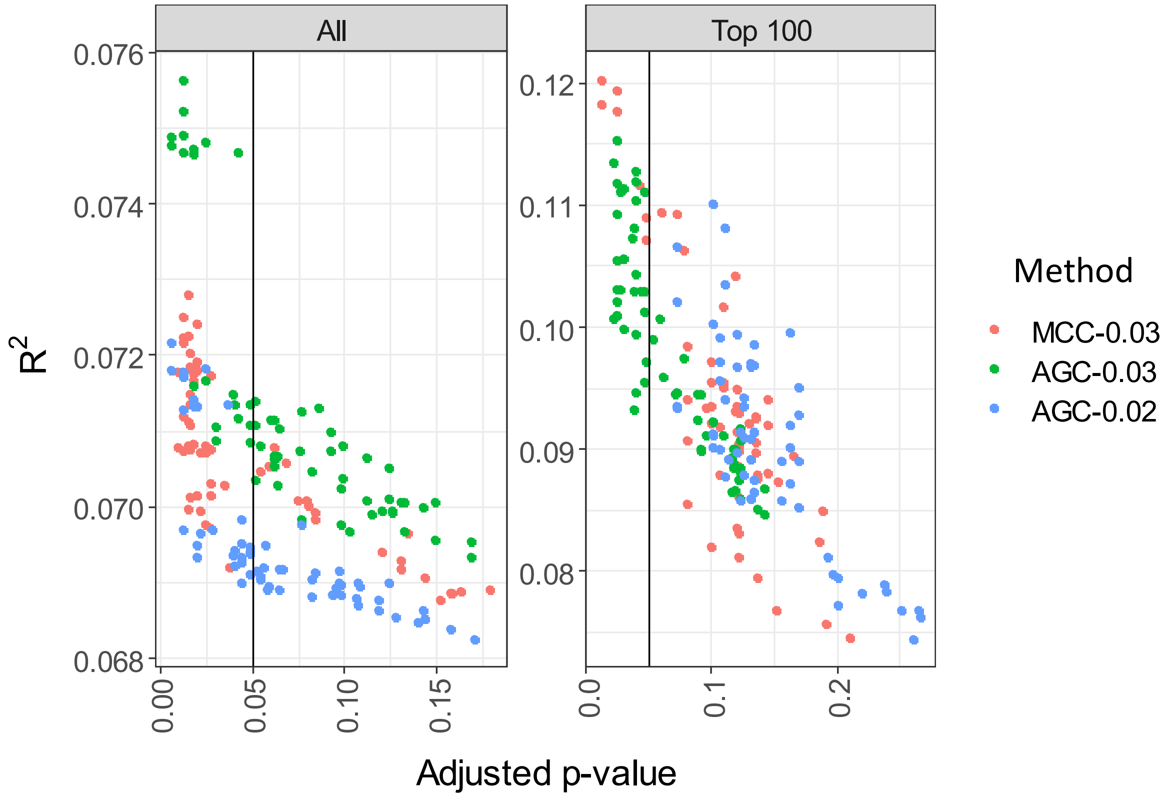

Figure S2. Effect size vs significance for all PERMANOVA trials on independently rarefied datasets for *nrfA*. Each point represents 1 PERMANOVA model.
